# Temperature Adaptation of Aquatic Bacterial Community Growth Is Faster in Response to Rising than to Falling Temperature

**DOI:** 10.1007/s00248-024-02353-8

**Published:** 2024-02-01

**Authors:** Erland Bååth, Emma S. Kritzberg

**Affiliations:** 1 Microbial Ecology, Department of Biology, Lund University, S-223 62 Lund, Sweden; 2 Aquatic Ecology, Department of Biology, Lund University, S-223 62 Lund, Sweden

**Keywords:** Temperature, Community adaptation, Bacterial growth, Lake water, Seasonal variation

## Abstract

Bacteria are key organisms in energy and nutrient cycles, and predicting the effects of temperature change on bacterial activity is important in assessing global change effects. A changing in situ temperature will affect the temperature adaptation of bacterial growth in lake water, both long term in response to global change, and short term in response to seasonal variations. The rate of adaptation may, however, depend on whether temperature is increasing or decreasing, since bacterial growth and turnover scale with temperature. Temperature adaptation was studied for winter (in situ temperature 2.5 °C) and summer communities (16.5 °C) from a temperate lake in Southern Sweden by exposing them to a temperature treatment gradient between 0 and 30 °C in ~ 5 °C increments. This resulted mainly in a temperature increase for the winter and a decrease for the summer community. Temperature adaptation of bacterial community growth was estimated as leucine incorporation using a temperature Sensitivity Index (SI, log growth at 35 °C/4 °C), where higher values indicate adaptation to higher temperatures. High treatment temperatures resulted in higher SI within days for the winter community, resulting in an expected level of community adaptation within 2 weeks. Adaptation for the summer community was also correlated to treatment temperature, but the rate of adaption was slower. Even after 5 weeks, the bacterial community had not fully adapted to the lowest temperature conditions. Thus, during periods of increasing temperature, the bacterial community will rapidly adapt to function optimally, while decreasing temperature may result in long periods of non-optimal functioning.

**Supplementary Information:**

The online version contains supplementary material available at 10.1007/s00248-024-02353-8.

## Introduction

Temperature is one of the most important environmental factors affecting aquatic organisms, including bacteria [1, 2]. On a global scale, bacteria have been found to grow in aquatic environments at temperatures comprised between 0 and 100 °C [1], but on a local scale, variation is of course much smaller. Still, seasonal variation in lake water can be substantial (> 20 °C), and even larger in small ponds [3]. Due to global change, annual mean temperatures [4] as well as temperature variation of lakes will increase, by increasing the magnitude and frequency of heatwaves [5, 6]. Given the key role of bacteria to energy and nutrient cycles and as degraders of organic material, predicting the effects of temperature change on bacterial activity is important in assessing global change effects.

**Fig. 1:**
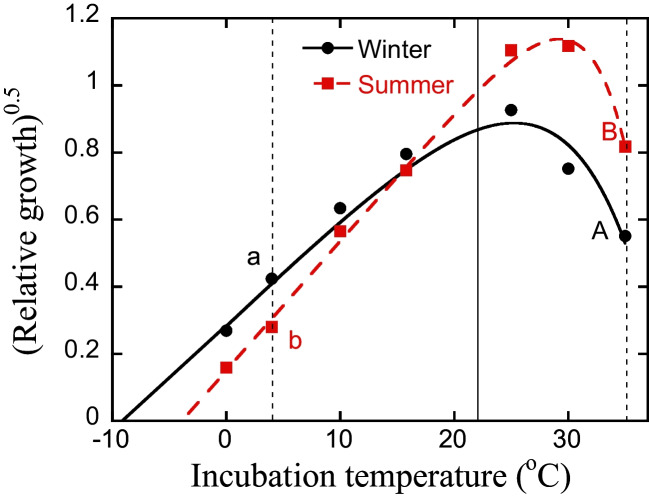
Hypothetical temperature response curves of relative bacterial growth of a winter and summer community plotted with a square root transformation. Community A (black circles and full line) is a low-temperature adapted community sampled in the winter (apparent minimum temperature for growth T_min_ = − 9 °C), and Community B (red squares and stippled line) is a high-temperature adapted community sampled in the summer (T_min_ = − 4.5 °C). Vertical thin stippled lines indicate temperatures used for calculating a temperature sensitivity index (SI, log growth at 35 °C/4 °C), where SI is log[(A/a)^2^] for community A and log[(B/b)^2^] for community B. SI for the high-temperature adapted community B will be higher than for the low-temperature adapted community A, and SI will correlate to T_min_. Thin full line indicates growth at 22 °C which was used as a measure of bacterial growth at a standardized temperature

Variation in water temperature affects bacterial growth rates within minutes of changing temperature [7]. Growth rates increase with increasing temperature up to the optimum temperature for growth (*T*_opt_), while lowering temperature will have the opposite effect [8]. This intrinsic temperature performance curve for pure culture bacterial growth can be well modelled by the square root model, also known as the Ratkowsky model [9], where the square root of growth will be linearly related to temperature below *T*_opt_. Using this model, the apparent minimum temperature (*T*_min_) can be determined by the *x*-axis intercept (Fig. 1). This model has also been shown to describe bacterial community growth in natural habitats, both in terrestrial and aquatic conditions [10–12]. *T*_min_ is the only variable needed to calculate the relative effect of temperature on bacterial growth below *T*_opt_ [10]. Furthermore, *T*_opt_ of natural bacterial communities is in most cases well above in situ temperatures, both in water and soil [10–12], and therefore only the part between *T*_min_ and *T*_opt_ will be of environmental relevance.

Apart from the immediate effect on bacterial growth rates, the environmental temperature will also select for bacteria better adapted to the in situ temperature, i.e., change the temperature adaptation of the bacterial community (defining community adaptation sensu [13, 14]). Community adaption is here defined as better performance of the community irrespective of the mechanism being due to species sorting or genetic changes within a species. Thus, environments with higher temperatures have communities adapted to high temperature and vice versa [10–12, 15–17]. This temperature adaptation can be estimated as changes in the cardinal temperatures, *T*_min_ and *T*_opt_, where especially the former will be informative on the relative effect of a sudden change in in situ temperature. On a global scale, *T*_min_ in aquatic environments has been suggested to vary from approximately − 17 to 0 °C (from Antarctic/Arctic conditions to warm sites with mean in situ temperatures around 0 °C and 30 °C, respectively [11]). A similar range has been found for soil [10, 16].

Bacterial communities may also adapt to seasonal variations in temperature. It was found that adaptation to seasonal variations of in situ temperature under a temperate climatic regime was faster in lake water than in soil, even though the temperature range of the two habitats was similar [12]. This was proposed to be due to a faster turnover of the bacterial community in water than in soil, resulting from higher exposure to predation. Laboratory experiments, where soils were kept at different temperatures, have also demonstrated that soil bacterial communities adapt only slowly to changes in temperatures. Soil communities kept at temperatures below *T*_opt_ did not adapt to temperature (i.e. *T*_min_ did not change) even after 1–2 months, while incubation above *T*_opt_, resulted in higher *T*_min_ [18–20]. Similar studies have not been performed for water habitats. Since faster turn-over of the bacterial community in lake water should allow faster community adaptation, one would predict that incubation at different temperatures would result in more rapid shifts in *T*_min_ in water than in soil.

The direction of the temperature change should also affect the rate of community adaptation. Increasing the temperature will increase community growth and turn-over rates, while lowering the temperature compared to in situ temperatures will decrease bacterial growth and thus turn-over rates. This was the suggested explanation of slower temperature adaptation of the bacterial community when moving soil up-slope (decreasing temperature) than when moving soil down-slop (increasing temperature) in a soil translocation experiment in an altitude gradient [13]. Similar results were also found in laboratory experiments with soil and peat [21, 22], suggesting that this is a general phenomenon, which should also be found in aquatic ecosystems.

To study to what extent, and how rapidly, adaptation of bacterial community growth to different temperatures occurred, short-term laboratory experiments with lake water were used. We hypothesized (1) that adaptation would be more rapid for lake communities than in similar laboratory studies with soil [18, 19], i.e., within days or weeks instead of months. To assess if there are differences in temperature adaptation in response to rising and sinking temperatures, we ran the experiment with communities sampled in winter (cold-adapted) and summer (warm-adapted), respectively (Fig. 1). Due to faster bacterial turn-over at higher temperatures, we hypothesized (2) that community adaptation would be faster in response to increasing the temperature compared to in situ conditions (for the cold-adapted winter community), than in response to decreasing the temperature (for the warm-adapted summer community). Bacterial growth was measured by leucine incorporation, and temperature adaptation of growth was assessed by estimating *T*_min_ and a temperature sensitivity index (SI, defined as log growth at 35 °C/growth at 4 °C).

## Materials and Methods

### Experimental Design and Sampling

Water from Lake Krankesjön (55°42′N, 13°28′E), Southern Sweden, was used. Lake Krankesjön is a shallow, nutrient-rich lake with a pH varying between 7.5 and 8.5 [23]. In situ temperature of the surface water varies annually approximately between 0 °C and 20 °C, and the seasonal variation in temperature adaptation of bacterial growth to this variation was earlier studied by Kritzberg and Bååth [12].

The experimental design consisted of incubating lake water at 7 different temperatures (0 to 30 °C) and then estimating temperature adaptation of the resulting bacterial communities over time by repeated short term measurement of growth at different temperatures (Fig. S1). Water was sampled at two occasions, once in the winter (28^th^ of February, 2.5 °C in situ temperature) and once in the summer (14^th^ of August, 16.5 °C in situ temperature). Water was collected just beneath the surface into 1-L plastic bottles. Subsamples of the unfiltered water sample were distributed into 50-ml Falcon tubes (45 ml in each), and duplicate tubes were placed in water baths at 0, 4, 12 (10 for the summer sample), 16, 20, 25, and 30 °C, respectively. This meant that for the winter sampling, most temperature treatments invoked a temperature increase relative to in situ conditions, while for the summer sampling, most temperature treatments invoked a temperature decrease relative to in situ. Bacterial growth was then monitored over time. Since it was expected that raising the temperature would result in more rapid temperature adaptation than decreasing the temperature, the summer experiment lasted for a longer period than the winter experiment (36 and 14 days, respectively, with an additional sample for the winter low temperature samples after 17 days due to expected lower rates of change at low temperatures). The water bath at 12 °C for the winter sample broke down after 7 days, and the sampling scheme for this treatment was then abandoned.

### Bacterial Growth Measurements and Calculations

At each sampling occasion, measurements of bacterial growth were made using leucine incorporation at three temperatures: 4, 22, and 35 °C. The measurements at 22 °C were used as a temperature standardized value for bacterial growth for all treatments, where immediate effects of changing temperatures were avoided (Fig. 1). As a proxy for temperature adaptation of the community, a temperature sensitivity index (SI) was calculated as log growth at 35 °C/growth at 4 °C, thus using one temperature above and one below *T*_opt_ (Fig. 1). This index has been shown to be a sensitive and robust indicator of community adaptation to temperature, correlating well to *T*_min_ [12, 17]. Using SI, there is also no need to specify any model of temperature sensitivity. Increasing SI indicates growth adaptation to higher temperatures of the bacterial community and vice versa.

Temperature adaptation of the bacterial community was also assessed by calculating *T*_min_, but only once after 14 days of experimental temperature treatments, for both the winter and summer experiment. Bacterial growth was then determined from incubations at 0, 4, 12 (10 for the summer experiment), 16, 20, 25, and 30 °C. *T*_min_ was calculated using the square root model (the Ratkowsky model [9]), where the square root of bacterial growth is linearly related to the incubation temperature below the optimum temperature for growth.

(1) √Growth = a*(T-T_min_) 

*a* is a slope variable, *T* is the incubation temperature, and *T*_min_ is the apparent minimum temperature for growth. *T*_min_ is determined by linear extrapolation as the crossing with the *x*-axis (see Fig. 1). To avoid introducing nonlinear parts around optimum temperature for growth, only incubation temperatures ≤ 22 °C were used for the calculation. Increasing *T*_min_ indicates that the bacterial community adapts to higher temperatures [10, 12].

Bacterial growth was estimated with the leucine (Leu) incorporation method [24, 25]. Subsamples of 1.5 ml of water were transferred from each Falcon tube to microcentrifugation vials. For the intended incubation temperature to be reached, the vials were pre-incubated in water baths: 2 h for 0 °C; 1 h for 4, 10, 17, 22, and 25 °C; and 30 min for 30 and 35 °C. Incubation began with the addition of 2 μl of 1-[4,5-^3^H]-Leucine (5.7 TBq mmol^−1^, Perkin Elmer, USA) and unlabeled Leu, resulting in a final Leu concentration of 275 nM. In order to achieve a more similar total incorporation of Leu irrespective of temperature, the incubation time was varied with incubation temperature [12, 26]. At 0 °C, incubation time was around 22 h; at 4 °C 6 to 7 h; at 10 °C 4 h; at 17 °C 2 h; at 22 °C 1.5 h; and at 25, 30, and 35 °C 1 h. Incubation was terminated by adding 75 µL of 100% trichloroacetic acid (TCA), resulting in a final concentration of 5% TCA, terminating growth. Non-incorporated Leu was washed away following the washing steps according to [27]. Finally, 1 mL of scintillation cocktail (Ultima Gold; PerkinElmer, USA) was added to the samples, and radioactivity was measured using a liquid scintillation counter (PerkinElmer Liquid Scintillation Analyzer, Tri-Carb 2910 TR). Obtained values are presented as relative growth, that is, ^3^H-Leu incorporation as DPM ml^−1^ h^−1^.

## Results

### Initial Temperature Adaptation of the Winter and Summer Community

The winter and summer samples differed in the initial temperature adaptation of the bacterial community. The winter community was adapted to cold conditions, as evidenced by a low SI (Sensitivity Index; SI, log growth at 35 °C/4 °C) around − 0.2, while the summer community was adapted to high temperature with an SI around 0.9. This is approximately equivalent to *T*_min_ of − 11 °C and − 4 °C for the winter and summer community, respectively.

### Temperature Adaptation of Bacterial Growth

**Fig. 2:**
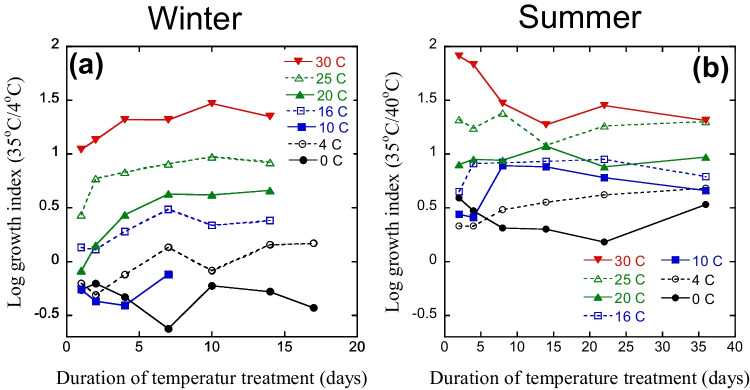
Temperature adaptation (expressed as the temperature sensitivity index (SI) for bacterial growth, log growth at 35 °C/4 °C) in lake water from different temperature treatments. The winter sample (**a**) was taken at *in situ* temperature 2.5 °C and summer sample (**b**) at in situ temperature 16.5 °C. Each data point is the mean of two samples. Note different time scales for (**a**) and (**b**)

The temperature adaptation of bacterial growth of the cold-adapted (winter) community changed very rapidly when kept at different temperatures (Fig. 2a). Initially SI was around − 0.2, but already after one day SI increased at higher treatment temperatures, indicating community adaptation. At 30 °C, SI was around 1 after one day, while for temperatures ≤ 20 °C, SI was still low, between − 0.2 and 0.1. Further changes in SI over time were then found at most temperatures, stabilizing at constant values after 7 days. The highest SI was found for 30 °C (almost 1.5), decreasing with decreasing treatment temperatures. Thus, already after 1 week at different temperatures, clear differences in temperature adaptation were found throughout the whole treatment temperature range. After 2 weeks (end of temperature treatment), the range of SI values was almost 2 units when comparing 0 °C and 30 °C.

The temperature adaptation of the summer communities in response to the different temperature treatments was slower than for the winter community. Only small changes in SI were found at low temperatures during the first days, while a larger change was seen in 25 and 30 °C treatments. Variations in SI were seen the first days, probably due to rapid changes in activity. After around 2 weeks, however, SI stabilized (Fig. 2b), and after 5 weeks, clear effects of treatment temperatures were found. SI for the high temperature treatments were similar for the winter and summer samples (around 1.5), while SI for the lowest treatment temperatures was higher for the summer samples, around 0.5 compared to < 0 for the winter samples (Fig. 2). The range in SI was thus only around 1 unit for the summer samples kept at the different temperatures for 5 weeks. Thus, even after 5 weeks at low temperatures, the bacterial community from the summer sample had not fully adapted to the low temperature conditions.

**Fig. 3:**
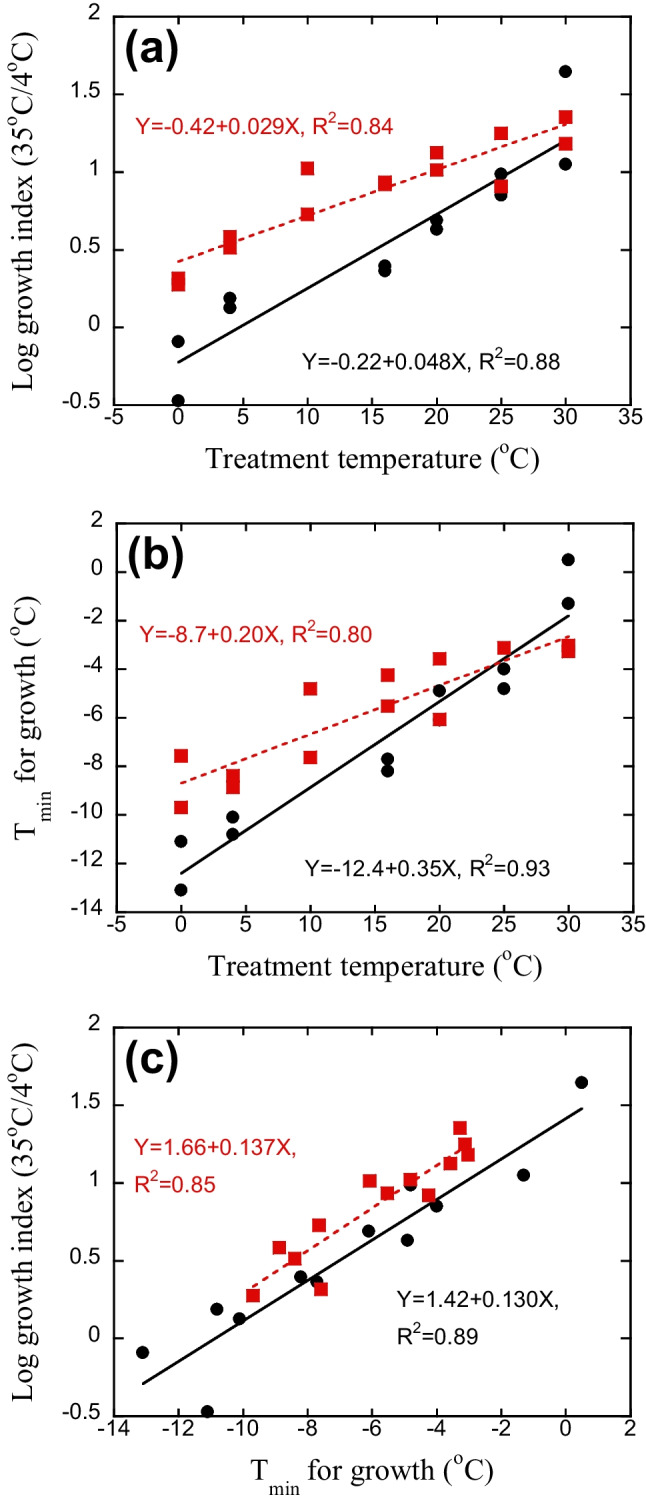
Temperature adaptation of bacterial growth after 14 days’ treatment at different temperatures, determined as **a** the temperature sensitivity index (SI) for bacterial growth (log growth at 35 °C/4 °C) and as **b** T_min_. **c** Correlations between the two measures of temperature adaptation of bacterial community growth. Black circles and full line denote the winter sample (in situ temperature 2.5 °C) and red squares and stippled line the summer sample (in situ temperature 16.5 °C)

A further comparison of the summer and winter samples was made by regressing temperature adaption after 14 days (SI and *T*_min_, the latter only determined at this time point) against treatment temperature (Fig. 3). For the winter sample, where temperature was increased compared to in situ conditions for almost all temperatures, SI varied between − 0.2 and 1.2 for samples kept between 0 and 30 °C, with a slope of 0.048 (Fig. 3a), and with similar slopes for earlier days (a mean slope of 0.052 for day 4 to 14). The variation in SI was smaller for the summer sample, where most of the treatment temperatures were lower or similar to in situ conditions. SI only varied between 0.4 and 1.3, with a slope of 0.029 at day 14 and with similar slopes at later time points (mean slope of 0.032 at day 22 and 36). Similar results, with less variation for the summer compared to the winter sample, were found for *T*_min_ (after 14 days, Fig. 3b). *T*_min_ for the winter sample varied between − 12 and − 2 °C (water kept at 0 to 30 °C), with a slope of 0.35 °C per degree of change in temperature. *T*_min_ of the summer sample only varied between − 9 and − 3 °C, with a slope of 0.20 °C per degree of change in temperature (Fig. 3b). The similar results for SI and *T*_min_ resulted in close correlations between these two measurements of temperature adaptation of the bacterial community (Fig. 3c), with no differences in the slope for the winter and summer community (*p* > 0.05).

### Temperature Standardized Bacterial Growth

**Fig. 4:**
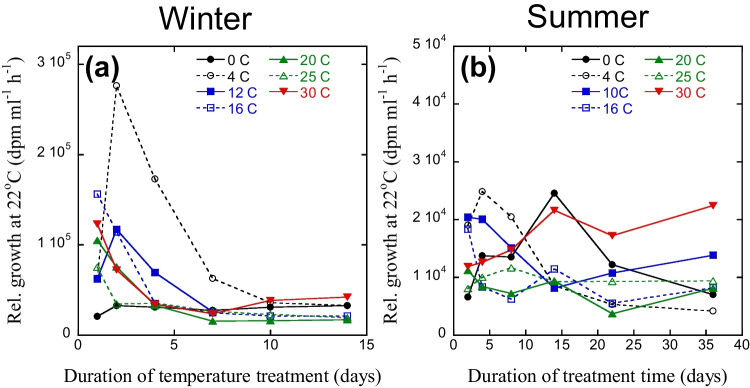
Standardized bacterial growth at 22 °C (Leu incorporation) in lake water sampled in winter (**a**) and summer (**b**) and then kept at different temperatures. Each data point is the mean of two samples. Note different time scales for (**a**) and (**b**)

Bacterial growth at a standardized incubation temperature (22 °C) initially increased in lake water sampled in the winter (Fig. 4a). This response was faster at higher treatment temperatures as reflected by the timing of the maximum growth at different incubation temperatures. For the 0 °C treatment, maximum growth was found after > 14 days, and thus growth increased throughout the experiment. At 4 and 12 °C, maximum growth was found after around 2 days, while for treatment temperatures ≥ 16 °C, maximum growth was after 1 day or less. The latter resulted in an apparent decreased growth until the end of the experiment for these treatments. After maximum growth was reached, the decrease in bacterial growth was also temperature dependent, with a faster decrease at higher treatment temperatures (Fig. 4a).

Differences were found in the development of growth at a standardized temperature over time also for the summer sample kept at different temperatures (Fig. 4b). At 0 °C, maximum growth was found after around 14 days, at 4 °C after 4 days, and at 10 °C after 1–2 days. At higher temperatures, maximum growth presumably occurred even sooner. The decreasing growth after maximum growth was also temperature-related, although for the 30 °C treatment, an increased growth was found throughout the duration of the experiment.

## Discussion

A prerequisite of the experiment was that the bacterial community sampled in the winter was adapted to low temperature conditions, while the opposite would be the case for the summer community. Seasonal variation in temperature adaptation of bacterial growth, estimated as *T*_min_, varied between − 4.5 °C (in the summer) and − 10.5 °C (winter) in a previous study including lake Krankesjön [12]. Estimating *T*_min_ from samples kept at approximately the in situ temperatures (Fig. 2) suggested a *T*_min_ for the winter and summer samples around − 11 °C and − 5 °C, respectively. Thus, both the absolute level and amplitude of the seasonal variation of *T*_min_ for bacterial growth in this lake were in line with the previous study.

Irrespective of the time of sampling the lake, the laboratory experiments resulted in community adaptation of bacterial growth within days in response to the new temperature regimes (Fig. 2). This was in contrast to earlier studies in soil, where such adaptation was not found even after to 2 months at temperatures differing with up to 20 °C [18–20]. The faster adaptation to changing temperatures in water than in soil was in accordance with our first hypothesis. A similar difference in the rate of community adaptation of bacteria between soil and aquatic environments to other environmental factors than temperature was recently suggested by Martiny et al. [28]. As for differences observed in seasonal variations in temperature adaptation between lake water and soil [12], our results could be explained by faster growth and turnover of the community in lake water compared to soil.

Already after 2 weeks, the effect of changing temperature on *T*_min_ for the winter community (changing with 0.35 °C per degree change in temperature) was similar to that found in the seasonal study (0.32 °C per degree change of in situ temperature [12]). It also falls close, albeit in the low range for water, to the suggested global effects of varying annual mean temperatures in soil and aquatic habitats (0.2 to 0.3 °C for soil [10, 17, 29]; 0.43–0.67 °C for water [11]). Thus, the bacterial winter communities had acquired the expected or close to the expected temperature adaptation at all treatment temperatures after 2 weeks. This was not the case with the summer community, which only changed with 0.20 °C per degree change even after 5 weeks’ temperature treatment. Raising the temperature (with increasing community turnover) thus resulted in more rapid community adaptation than lowering the temperature (decreasing turnover), in accordance with our second hypothesis. Even if the increase of the winter community (almost 28 °C) was larger than the decrease of the summer community (16 °C), temperature adaptation of the winter community would be more rapid than the summer community also if only including the results from 0 to 20 °C treatments (Fig. 3). The difference of increasing and decreasing temperature on the rate of temperature adaptation has previously been found for soil, e.g., after translocation of soil in a temperature (elevation) gradient [13] and in laboratory experiments with peat and soil [21, 22]. This differential effect thus appears to be a general phenomenon, underlining that it is not only the extent and amplitude of the temperature change that will determine the rate of community adaptation but also the direction of this temperature change. Consequently, we would expect different dynamics in the summer-autumn, with decreasing in situ temperatures, and then in winter-spring with increasing temperatures, especially in aquatic systems. However, to detect such seasonal differences in adaptation rate, a much higher frequency of sampling than used in the seasonal study of Kritzberg and Bååth [12] would be required.

Keeping water in the laboratory in small containers may influence bacterial growth by the so-called bottle effect, increasing growth compared to natural conditions (e.g., [30]). A similar effect is not found when incubating soil [31]. Thus, the difference in adaptation rate in response to a temperature change between water and soil, with changes within days or months, respectively, may to some extent be due to incubation conditions. However, “bottle effects” are small or not always found with the experimental setup here (around 50 ml water [30, 32]) suggesting that differences in growth rate and turnover of the biomass were the main explanation for the difference between water and soil. More importantly, any “bottle effect” would be similar for all treatments, as concluded in studies on temperature effects on bacterial diversity [33]. Thus, “bottle effects” cannot explain different adaptation rates between increasing or decreasing the temperature.

The results of the present laboratory study support our main hypothesis of differences in rates of temperature adaptation in soil and water. Thus, it will be important to include seasonal variations in temperature adaptation of bacterial growth in water habitats, while this will be less important in soil. Furthermore, the rate of adaptation will be different depending on increasing or decreasing temperature, even with the same absolute change in temperature amplitude. Thus, one would expect temperature adaptation to be more rapid in spring, with increasing temperatures, than in autumn with decreasing temperatures. A corollary of this would be that during falling temperature conditions in the autumn, there will be longer periods with not fully adapted and thus not perfectly functioning communities, compared to spring conditions. This is similar to the situation for desert soils with large fluctuations in seasonal temperatures [16].

An increase in temperature of 1 °C in cold habitats will result in a relatively larger change in the growth rate of a community than a 1 °C increase in a warm habitat, i.e., Q_10_ (relative rate at temperatures with 10 °C difference) decreases with increasing temperature [10]. However, the absolute effect on growth will be higher in a warm habitat, since absolute growth is faster at high temperatures. Given that the rate of adaptation depends on growth rate and turn-over of the bacterial community, one would thus predict a 1 °C temperature rise in cold habitats to result in a slower rate of community adaption, than a 1 °C increase in warmer habitats. Furthermore, this difference will not only apply to the temperature adaptation of the bacterial community, but also community adaptation to other environmental factors, like pH [34] or pollutants [35, 36]. Thus, the temperature has to be taken into account when studying temperature adaptation of the bacterial community, but also community adaptation to other environmental factors expected to change.

### Supplementary Information

Below is the link to the electronic supplementary material.

Fig. S1 Experimental design (TIFF 590 KB)

## Data Availability

Primary data are available upon request from the corresponding author.
