# Supplementary figures and images for: Temperature Adaptation of Aquatic Bacterial Community Growth Is Faster in Response to Rising than to Falling Temperature
Source: Microb Ecol. 2024 Feb 1;87(1):38. doi: 10.1007/s00248-024-02353-8 (PMC10830665; doi:10.1007/s00248-024-02353-8)

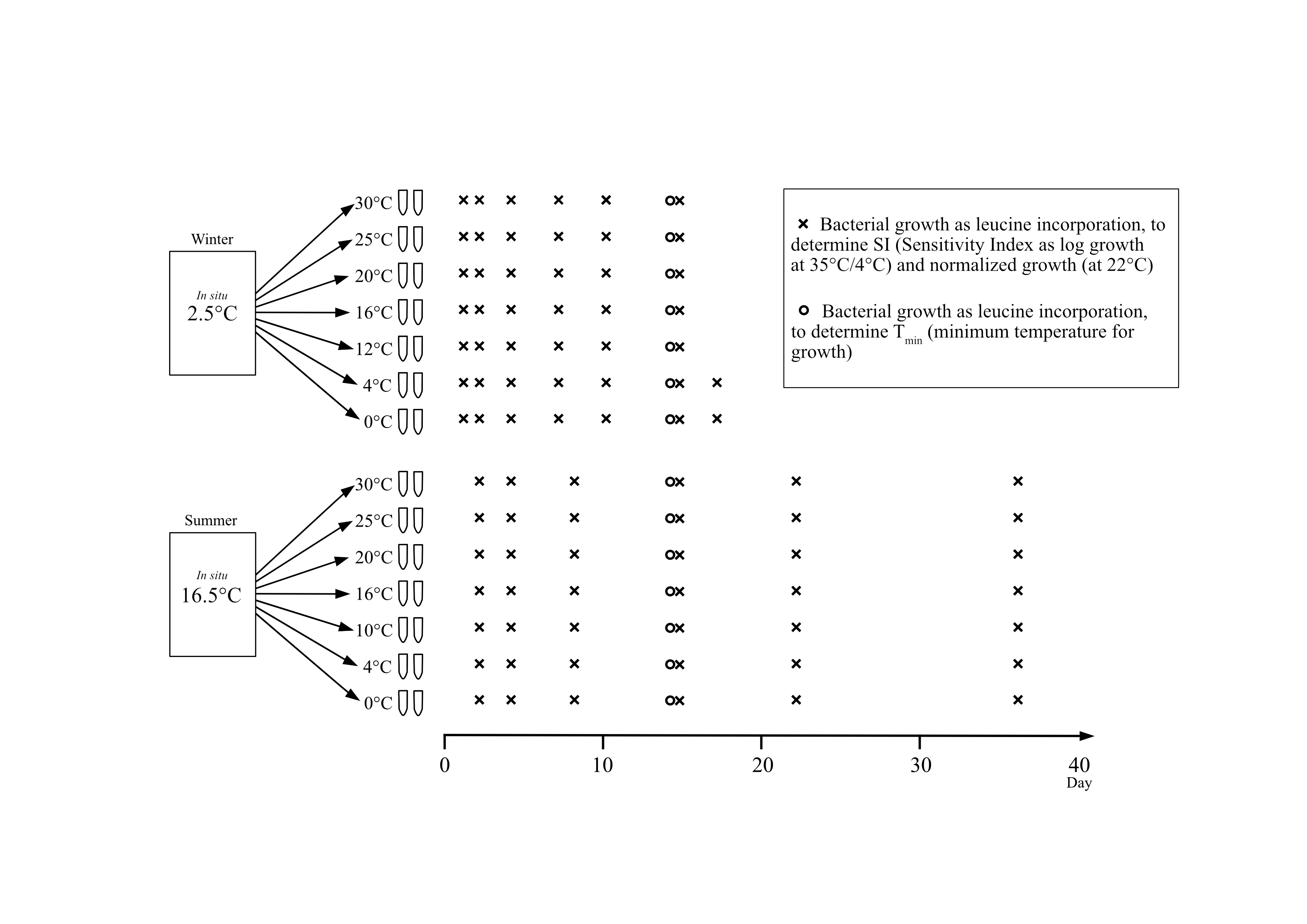

Supplement: Supplementary file 1 — Experimental design (TIFF 590 KB) [file 248_2024_2353_MOESM1_ESM.tiff]
